# Immunomodulation by AZD1656 reverses cardiac dysfunction, metabolic remodelling and reduces infarct size in type 2 diabetic cardiomyopathy

**DOI:** 10.1101/2025.04.10.648128

**Published:** 2025-04-11

**Authors:** Stephanie Anderson, Anja Karlstaedt, Jianmin Chen, Caroline E. O’Riordan, Michael R. Barnes, Zorana Štaka, Lauren J. Albee, Conor Garrod-Ketchley, Sanushi Thamasha Dambure Vithanachchi, Hiran A. Prag, Filip Cvetko, Christoph Thiemermann, Andrew JM. Lewis, Michael P. Murphy, David M. Smith, Sian M. Henson, Damian J. Tyler, Dunja Aksentijevic

**Affiliations:** 1 Department of Physiology, Anatomy and Genetics, University of Oxford, Oxford, UK; 2 Department of Cardiology, Smidt Heart Institute, Cedars-Sinai Medical Center, Los Angeles, USA; 3 William Harvey Research Institute, Bart’s and The London Faculty of Medicine and Dentistry, Queen Mary University of London, London, UK; 4 School of Cardiovascular Science and Medicine, The Rayne Institute, St Thomas Hospital, King’s College London, London, UK; 5 MRC Mitochondrial Biology Unit, University of Cambridge, Cambridge, UK; 6 Faculty of Electrical Engineering, University of East Sarajevo, East Sarajevo, Bosnia and Herzegovina; 7 Emerging Innovation Unit, Discovery Sciences, R&D, AstraZeneca, Cambridge, UK

**Keywords:** type 2 diabetic cardiomyopathy, inflammation, metabolic remodelling, cardio-immunology, type 2 diabetes

## Abstract

Type 2 Diabetes (T2D) can lead to diabetic cardiomyopathy (dbCM), which is characterised by chronic, systemic inflammation, disrupted metabolism and impaired cardiac function. However, whether cardiac inflammation is present in dbCM and causally linked to metabolic remodelling remains unknown. AZD1656 (AZD), an activator of glucokinase, was postulated to provide glycaemic control in T2D by acting on in the pancreas and liver. However, AZD failed to control hyperglycaemia in clinical trials. Nevertheless, testing of the drug in COVID-19 T2D patients as part of the ARCADIA trial indicated an immunomodulatory effect. Therefore, we used the db/db mouse model of dbCM and an integrated in vivo and ex vivo experimental approach to examine the effects of AZD on cardiac functional and metabolic disturbances and inflammation. 20-week db/db mice displaying the features of human dbCM (obesity, hyperglycaemia and diastolic dysfunction) treated for six weeks with AZD showed improved metabolic remodelling, attenuated diastolic dysfunction, reduced infarct size and improved functional post-ischemic recovery compared to untreated dbCM, alongside an improved cardiac immunophenotype, including reduced T cell-mediated fibrosis and B cell infiltration. Therefore, targeting of immunometabolism may offer a new therapeutic approach to treat cardiac dysfunction and metabolic dysregulation and reduce infarct size in dbCM.

## Introduction

Diabetic cardiomyopathy (dbCM) is a complication of type II diabetes (T2D), characterized by impaired cardiac function, disrupted metabolism, systemic inflammation and consequently increased incidence of ischemic heart disease and heart failure^1,2^. The dbCM heart is metabolically inflexible relying heavily on fatty acid (FA) oxidation^3^. This loss of metabolic flexibility means the dbCM heart is chronically energetically impaired^4^. Whilst metabolic derangements are a well-established risk factor for a worsening prognosis in dbCM, currently there are no therapeutic agents specifically designed to target cardiac metabolism in dbCM.

Glucokinase and hexokinase catalyse glucose phosphorylation to glucose-6-phosphate in the first step of glycolysis. While hexokinase is found in most tissues, glucokinase is primarily found in the pancreatic β-cells, the liver, and a subset of T cells. In these tissues, glucokinase acts as a glucose sensor and regulates glucose-stimulated insulin secretion and hepatic glucose uptake. As such, activating glucokinase is an attractive target for the treatment of T2D.

AZD1656 (AZD) is a specific activator of glucokinase with >100-fold selectivity over hexokinase and was expected to provide effective glycaemic control in T2D through its activity in pancreas and liver^5^. However, in twenty-five clinical trials involving 900 type 2 diabetic patients^6,7^ AZD failed to fulfil its primary endpoint of controlling hyperglycaemia, only improving glucose levels for up to 4 months. More recently, AZD was tested in COVID-19 patients with T2D^8^ as part of the ARCADIA trial. This trial outcome suggested a beneficial effect of AZD as the treatment decreased deaths whilst extensive patient phenotyping indicated an immunomodulatory effect of the drug^8^. Specifically, patient T-cell immunophenotyping suggested that AZD-treated T2D COVID-19 patients had a less pro-inflammatory immune response and a better adaptive immune response than those treated with placebo^8^.

Chronic inflammation is a hallmark of T2D, accompanied by increased circulating levels of highly inflammatory senescent T-cells^2,9,10^. Proinflammatory stimuli such as systemic metabolic stress in T2D (hyperinsulinemia, hyperlipidaemia, hyperglycaemia, oxidative stress) may trigger T cell-mediated autoimmunity leading to chronic inflammation in the heart^1^. Despite this, many anti-inflammatory agents have failed to improve cardiac function in clinical trials as they mostly target innate immunity and acute inflammation^1,11^. Systemic hyperlipidaemia characteristic of T2D has also been shown to promote the differentiation of pro-inflammatory effector memory T-cells (Tem), while reducing the number of regulatory T-cells, essential for immune homeostasis^12^. In chronic metabolic stress states such as T2D, high circulating fatty acids have been shown to drive the differentiation of CD4+ Tem and migration into non-lymphoid tissues thus promoting inflammation by pro-inflammatory T-cell cytokine release and systemic inflammasome activation^1,12^. However, whether systemic T-cell-mediated inflammation in T2D leads to myocardial inflammation and whether it impacts metabolic derangement in dbCM remains unknown.

Due to the potential immuno-modulatory effect of AZD, we set out to test the immune-regulatory potential of AZD during cardiac metabolic and functional derangement in T2D. We show that a 6-week AZD drug intervention in the db/db mouse model of dbCM attenuated diastolic dysfunction, reversed metabolic remodelling and reduced infarct size versus untreated dbCM. The beneficial cardiometabolic effects of AZD treatment were not driven by systemic metabolic alterations in the liver, muscles or adipose tissue but AZD did reduce cardiac inflammation and fibrosis.

AZD also normalizes myocardial gene expression including those mediating inflammation as well as governing key pro-survival and metabolic pathways (i.e HIF-1α, Nrf2, sirtuin, PPARα, apoptosis). Consequently, AZD treatment may represent a new therapeutic option for dbCM by targeting immunometabolism to reduce cardiac inflammation and improve cardiac remodelling.

## Materials and Methods

### Animals

Commercially available type 2 diabetic mice (db/db mouse, The Jackson Laboratory homozygous BKS.Cg-Dock7^m^ +/+ Lepr^db^/J, male, Charles River, Italy) were purchased at 8 weeks with corresponding lean littermate controls (heterozygous Dock7^m^ +/+ Lepr^db^, Charles River, Italy). Animals were kept under pathogen-free conditions, 12h light–dark cycle, controlled temperature (20–22°C), and fed chow and water *ad libitum*. Circulating glucose (tail sample, Accu-Check, Roche) and body weight were monitored weekly. This investigation conformed to UK Home Office Guidance on the Operation of the Animals (Scientific Procedures) Act, 1986.

### AZD1656 drug treatment protocol

**Figure 1.**
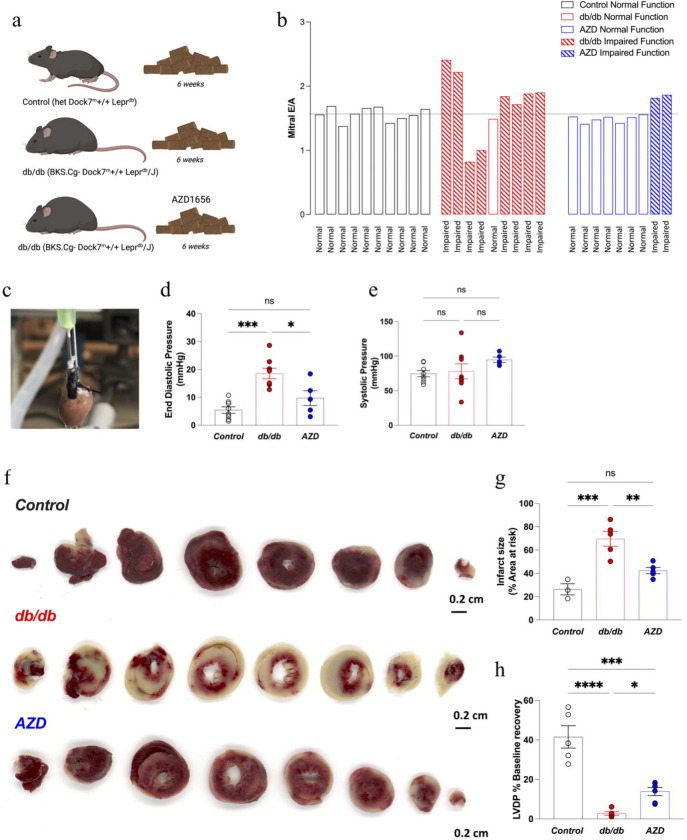
AZD treatment attenuates diastolic dysfunction and reduces infarct size in dbCM a) Graphical summary of the AZD treatment protocol. Mice were fed nutritionally standardised and matched Teklad Standard Base Diet (Envigo) with the addition of AZD1656 to the diet used for db/db treatment group b) In vivo echocardiography assessment of mitral valve E/A, degrees of diastolic dysfunction, classification based on previously validated criteria in ^20,21^. Classification also summarized in Methods. Dotted line represents control E/A average. c) representative Langendorff perfused heart d) e)Langendorff perfused heart function data (Control n=9, db/db n=9, AZD n=6) f) Representative TTC stained cardiac cross sections post 20 min total global ischemia and 2 hr reperfusion used for quantification of the infarct size. g) Quantification of infarct size post I/R injury (db/db, AZD n=5/group, control n=3) h) Improved LVDP recovery post ischemia in Langendorff perfused hearts (Control n=5, db/db n=5, AZD n=6) f) *P<0.05, ***P<0.01 by ANOVA

AZD1656 (AZD, Astra Zeneca, UK) is a selective glucokinase activator. No significant clinical effects nor specific toxicology risks other than hypoglycaemia were noted in clinical trials^13^. The db/db and lean controls were divided into 3 groups at 13 weeks of age: Group I - lean controls, Envigo diets control diet (2019 Teklad Global 19% Protein Rodent Diet, irradiated, Teklad Custom Diets, Envigo), Group II - db/db, Envigo diets control diet and Group III - db/db, AZD1656 diet (30 mg/kg body weight/day, Envigo Specialist Diets, USA; drug dosing based on^14,15^), Figure 1a. Diets were fully matched in terms of nutritional standardisation and food intake was not affected by dietary drug incorporation. Animals (study total: Group I - Controls n=56, Group II - db/db n=55, Group III - AZD n=49) were kept on the feeding protocol from 14 until 20 weeks of age when the study reached endpoint.

### Body Composition analysis

Body composition analysis was carried out by non-invasive magnetic resonance relaxometry using an EchoMRI^™^ Body Composition Analyser E26–348-MT (Houston, Texas, USA). Accumulation factor was for extra-high precision (3×) resulting in a scan time of approximately 2.5 min.

### In vivo Assessment of left ventricular systolic and diastolic function

#### CINE Magnetic Resonance Imaging

For the assessment of systolic function, mice were imaged in a 7T MRI instrument (Agilent, USA) using CINE magnetic resonance imaging, as previously described^16^. Eight to 10 short-axis slices (slice thickness, 1.0 mm; matrix size, 256 × 256; field of view, 25.6 × 25.6 mm; echo time/repetition time, 0.3/4.6 ms; flip angle, 30°; and number of averages, 4) were acquired with a gradient echo, fast low-angle shot sequence^17^. Left ventricular volumes were derived using the freehand draw function in ImageJ (National Institutes of Health). For each heart, left ventricular mass, ejection fraction, stroke volume, and cardiac output were calculated.

#### Echocardiography

M-mode and Doppler echocardiography were performed in control, db/db and AZD mice at the end of the drug feeding protocol as previously described^18,19^. Anaesthesia was induced with 3% isoflurane and maintained at 0.5 to 0.7 % for the duration of the procedure. Before assessment of cardiac function, fur was removed from the chest area to allow accurate assessment of cardiac function and mice were allowed to stabilize for at least 10 minutes. Body temperature was maintained at 37 °C. During echocardiography, the heart rate was measured from electrocardiogram and were kept consistent between experimental groups. Echocardiography images were recorded using a Vevo- 3100 imaging system with a 40-MHz linear probe (VisualSonics, Toronto, Canada). Diastolic transmitral left ventricle (LV) inflow images were acquired from apical four-chamber views using pulsed-wave Doppler to calculate early (E) and late (atrial, A) peak filling blood flow velocities and E-wave deceleration time. The E/A ratio represents the ratio of E wave to A wave. The sample volume was positioned at the tip of the mitral valve leaflet in the mitral valve annulus, the ultrasound beam was parallel with the direction of blood flow to record maximal transmitral flow velocities. Left atrial area was measured in apical four-chamber views, borders of the left atrium were traced just before mitral valve opening at end ventricular systole. *In vivo* echocardiography assessment of mitral valve E/A, degrees of diastolic dysfunction, classification were based on.^20,21^ Normal diastolic function was defined as E/A 1.0–1.5, diastolic dysfunction as E/A<1.0 (impaired relaxation) and E/A >2.0 (restricted grade III and grade IV). ^20,21^ Cardiac function was assessed blinded to the phenotype.

#### Hyperpolarized Magnetic Resonance Spectroscopy

For the *in vivo* assessment of cardiac metabolism, hyperpolarized magnetic resonance spectroscopy was used to monitor the down-stream fate of hyperpolarized [1-^13^C]pyruvate as previously described^22^. Experiments were performed between 7 and 11 A.M. when mice were in the fed state. A total of 40 mg [1-^13^C]pyruvic acid (Sigma-Aldrich) doped with 15 mmol/L trityl radical (OXO63; GE Healthcare) and 3 mL Dotarem (1:50 dilution; Guerbet) was hyperpolarized in a prototype polarizer, with 30–60 min of microwave irradiation^23^. The sample was subsequently dissolved in a pressurized and heated alkaline solution, containing 2.4 g/L sodium hydroxide and 100 mg/L EDTA dipotassium salt (Sigma-Aldrich), to yield a solution of 80 mmol/L hyperpolarized sodium [1-^13^C]pyruvate with a polarization of ~30%. A total of 200 μL was injected over 10 s via the tail vein. ^13^C MR pulse-acquire cardiac spectra were acquired over 60 s following injection of hyperpolarized [1-^13^C]pyruvate (repetition time, 1 s; excitation flip angle, 15°; sweep width, 13,021 Hz; acquired points, 2,048; and frequency centred on the C1 pyruvate resonance)^22^. The ^13^C label from pyruvate and its metabolic products was summed over 60 s from the first appearance of pyruvate and fitted with the AMARES algorithm in jMRUI^24^. Data are reported as the ratio of metabolic product to the [1-^13^C]pyruvate signal to normalize for differences in polarization and delivery time.

#### Langendorff heart perfusions

Mice were terminally anesthetized, hearts rapidly excised, cannulated and perfused as a standard Langendorff preparation as previously described^25^. Krebs-Henseleit (KH) perfusion buffer was continuously gassed with 95% O2/5% CO2 (pH 7.4, 37 °C) containing (in mM): NaCl (116), KCl (4.7), MgSO4.7H2O, KH2PO4 (x), (1.2), NaHCO3 (25), CaCl2 (1.4), and enriched with metabolites [glucose (11), intralipid 0.4mM, 1 sodium L-lactate; 0.1 sodium pyruvate; 0.5 L-glutamic acid monosodium salt monohydrate; 5 mU l^−1^insulin (NovoRapid insulin, Novo Nordisk, Denmark),] and paced at 550bpm via epicardial silver wire electrodes placed at the apex of the left ventricle and the right atrium. The impact of 1μM AZD^26^ on heart function and metabolism was assessed in unpaced hearts perfused using hyperglycaemic (11mM) crystalloid KH buffer prepared in amber glassware immediately prior to the experiment. At the end of each experiment, hearts, liver, adipose tissue and muscle (gastrocnemius and soleus) were immediately freeze-clamped using Wollenberger tongs for metabolic profiling.

Myocardial infarct size was quantified as described before^27^. In brief, after 20 min of equilibration, Langendorff crystalloid KH buffer perfused hearts were subject to 20 min of global normothermic ischemia (37°C) and 2 hours of reperfusion (37°C). At the end of the protocol, hearts were perfused for 10 mins with 3% triphenyltetrazolium chloride (TTC) in KH Buffer followed by 10 min incubation in 3% TTC-KH. Tissue was sectioned (mouse heart gauge, Zivic instruments, USA) and infarct field was quantified using ImageJ Software (National Institute of Health).

#### Metabolomic profiling

Snap frozen and pulverized tissue [heart, liver, skeletal muscle(gastrocnemius+soleus), adipose tissue] was analyzed using ^1^H NMR high resolution spectroscopy as previously described^28,29^. Non-targeted lipid profiling of the cardiac tissue was performed by LC-MS MS by School of Chemical and Physical Sciences Mass Spectrometry Laboratory Services. Annotation of lipid species to lipid classes and categories was conducted using Lipidmaps^30^. MS intensities were then used to weight the molecular fatty acyl (FA) contributions and calculate class wise and total FA profiles.

#### In silico analysis of cardiac metabolism

In silico simulations were performed using the metabolic network of the cardiomyocyte, CardioNet^25^. Mathematical modelling has previously been used to study the dynamics of cardiac metabolism in response to stress, and CardioNet has been successfully applied to identify limiting metabolic processes and estimate flux distributions^25,31,32^. Optimization problems were defined with the objective to minimize the total sum of fluxes through the CardioNet metabolic network^33^. Simulations for control, db/db or AZD groups were based on the assumption that cardiomyocytes seek to maintain a certain ATP provision to sustain cardiac contractile function alongside the synthesis of macromolecules, including structural proteins and phospholipids for membranes^33^. We included the intracellular metabolite concentrations of 23 metabolites: NADH, formate, ATP/ADP pool, fumarate, glucose, (phospho)creatinine, glycine, taurine, carnitine, (phospho)choline, acetyl-carnitine, aspartate, glutamine, glutamate, succinate, acetate, alanine, lactate, valine and (iso)leucine. Furthermore, we constrained extracellular metabolites that could be taken up from the blood, including glucose, lactate, cholesterol and free fatty acids. Linear programming was solved using the GUROBI solver (version 9.1.2 build v9.1.2rc0, Linux64)^34^. Details of all reactions and their metabolic subsystems used for CardioNet analysis were as classified in the Kyoto Encyclopedia of Genes and Genomes database.^35^

#### Tissue extraction and digestion for fluorescence-activated cell sorting

To generate leukocyte single cell suspensions for characterisation of immune cell populations, hearts were isolated from mice and digested. Mice were euthanised using an overdose of anaesthesia with 5 % isoflurane in 2 L/min O_2_. Cessation of pedal and corneal reflexes were checked and death was confirmed by cervical dislocation. Cardiac tissue suspensions were prepared by perfusing hearts with cold HBSS for 5 min prior to removing the atria and mincing the ventricular tissue that was digested in collagenase I (Worthington Laboratories, C1639, 450 units/mL), collagenase XI (Worthington Laboratories, C7657, 125 units/mL), DNase 1 (Worthington Laboratories, D4527, 60 units/mL) Hyaluronidase (Sigma Aldrich, H3506, 60 units/mL) and 20mM Hepes in PBS for 20 minutes at 37°C with gentle agitation (Thermomixer, 750 rpm). Samples were passed through a 70 μm cell strainer, rinsed with 10 mL cold 2% FBS/ PBS and centrifuged at 400g for 10 min at 4°C. Supernatant was removed and pellets resuspended in 5 mL red blood cell lysis buffer (BioLegend) and incubated on ice for 10 min with occasional agitation after which 10 mL cold 2% FBS/ PBS was added to neutralise the lysis. Samples were then centrifuged for 8 min at 4°C and 400g, supernatant was removed and cells were resuspended and incubated with FC-block (BioLegend 101320, 1 μL per 1 × 106/ mL cells). Cells were then washed again and resuspended in PBS ready for counting and antibody staining.

#### Flow cytometry

Cells isolated from hearts were resuspended (~10^7^/ml) and incubated for 30 minutes at room temperature with fluorochrome-conjugated antibodies (Supplementary Table 1) in 100 μl of flow cytometry buffer made of PBS containing 0.1% sodium azide (SigmaAldrich) and 1% FBS. For intracellular marker staining, cells were fixed and permeabilized for 30 minutes at 4^o^C using fixation/permeabilization kit (eBioscience), washed in 1X permeabilization buffer (eBioscience) and stained with fluorochrome-conjugated antibodies in 1x permeabilization buffer for 30 minutes at 4°C. A final wash with 1x permeabilization buffer was performed, centrifuged and resuspended in 200μl of flow cytometry buffer. Alternatively, cells were fixed (Fix/Perfm kit, BioLegend 426803) and stored at 4°C. Cell viability was assessed using incubation with viability dyes (Supplementary Table 1). Samples were analysed on FACSAriaIII (BD Biosciences) running FACSDiVa v.8.0 software (BD Biosciences, Dunn School of Pathology, University of Oxford). CD3 beads (Miltenyi, UK) were routinely used to calibrate the cytometer. Single stain and fluorescence minus one control were acquired for compensation and precise gating (Supplementary Fig1 gating strategy). Compensation was automatically calculated, and samples analyzed using FlowJo software (version 10, FlowJo LLC, Oregon, USA).

#### Histology

Hearts were collected into buffered formalin (Sigma Aldrich). All histological processing was carried out by the Bart’s Cancer Institute Histology Core Facility. For the assessment of cardiac fibrosis, paraffin embedded sections were stained using masson trichrome staining. Stained cardiac sections were scanned using a Nanozoom panoramic scanner (40x). Images (whole heart sections) were analysed using ImageJ (National Institute of Health).

#### Plasma analysis

Blood samples were collected at the time of experimental endpoint into heparinized tubes. Plasma biochemical profiling was carried out by the MRC Mouse Biochemistry Laboratory (Addenbrookes NHS Hospital, Cambridge). Circulating plasma cytokines were assessed using XXL mouse cytokine array kit (Biotechne, USA) as described before^36^.

#### RNA sequencing and Bioinformatic analysis

RNA extracted from the snap-frozen hearts was analysed by massive analysis of cDNA End (MACE-Seq). Rapid MACE-seq was used to prepare 3’ RNA sequencing libraries. Samples of 100 ng of DNA-depleted RNA were used for library preparation, using the Rapid MACE-Seq kit (GenXPro GmbH, Germany). Fragmented RNA underwent reverse transcription using barcoded oligo(dT) primers containing TrueQuant unique molecular identifiers, followed by template switching. PCR amplified libraries were purified by solid phase reversible immobilization beads and subsequent sequencing was performed using the Illumina platform NextSeq 500. Unprocessed sequencing reads were adapter-trimmed and quality-trimmed using Cutadapt (version 3.4,^37^). Deduplication based on UMIs (Unique Molecular Identifier) was performed using in-house tools. FastQC (0.11.9, ^38^) was used to assess the quality of sequencing reads. MultiQC (version 1.16,^39^) was used to create a single report visualising output from multiple tools across many samples, enabling global trends and biases to be quickly identified. MACE-Seq was annotated, reads quantified and p values for differential expression generated by GenXPro (Frankfurt, Germany).

Visual representations of the differentially expressed gene (DEG) dataset were performed using the Python programming language (version 3. 9.7) as well as libraries and packages, including: Matplotlib (version 3.4.3) for data visualization, Pandas (version 1.3.4) for data management, NumPy (version 1.20.3) for numerical computations, and Jupyter Notebook (version 6.4.5) for interactive code development. The functional enrichment analysis was performed using g:Profiler (version e108_eg55_p17_0254fbf) with g:SCS multiple testing correction method applying significance threshold of 0.05^40^. To determine enriched pathways and ontologies in all analysis comparisons, both Ingenuity Pathway Analysis (IPA; Ingenuity^®^ Inc, Redwood city, CA) and Metascape comparison analysis (https://metascape.org)^41^ was performed on all genes with p<0.05. Comparisons were db/db_vs control (2740 DE genes) and AZ_vs_db/db (1271 DE genes). Both metascape and IPA utilise hypergeometric tests and Benjamini-Hochberg p-value correction to identify all ontology and pathway terms that contain a greater number of genes in common with an input list than expected by chance^41^.

#### Data analysis and statistics

Data are presented as mean ± SEM. Comparison between groups were performed by Student’s t-test (Gaussian data distribution), two-way analysis of variance (ANOVA) with Bonferroni’s correction for multiple comparison and one-way ANOVA using Bonferroni’s correction for multiple comparisons where applicable. Normality of data distribution was examined using Shapiro–Wilk’s normality test. Statistical analysis was performed using GraphPad Prism (v9) software. Data analysis and visualization was conducted using R Studio (version 2022.12.0 Build 353). Partial Least-squares Discriminant Analysis (PLS-DA) was conducted using R package *mdatools*. Principal Component Analysis (PCA) was conducted with normalized intensities lipidomics data using the PCA function included in the *factoextra* package.

Flux estimations (CardioNet) were compared between experimental groups using Wilcoxon rank sum test and adjusted p-values were computed using the Bonferroni correction. Differences were considered significant when P < 0.05.

## Results

### AZD treatment improves diastolic function in the diabetic heart and offers protection from IR injury.

The impact of 6-weeks of AZD treatment (Figure 1a) on cardiac function in dbCM was assessed *in vivo* by CINE MRI and echocardiography (Table 1). As has previously been noted^42^, no reduction in systolic function was observed in untreated db/db mice, with a small but significant alteration in ejection fraction observed alongside an unaltered cardiac output (Table 1). There was no evidence of pulmonary congestion or any increase in overall heart weight in db/db animals (Table 1). Nevertheless, db/db mice exhibited various degrees of diastolic dysfunction (Figure 1b) typical for dbCM. Deceleration time was increased in db/db mice further indicative of the development of diastolic dysfunction and LV stiffness (Table 1). Cine MRI assessment (Table 1) identified unaltered end diastolic volume and decreased end systolic volume. *Ex vivo* functional assessment by Langendorff perfusion (Figure 1d) further confirmed diastolic dysfunction in db/db mice.

Treatment with AZD enhanced *in vivo* cardiac function as improvements were noted in diastolic function (Figure 1b), myocardial performance index (Table 1), the doppler flow-assessed pulmonary artery VTI, pulmonary artery peak velocity (Table 1) and ascending aorta peak velocity (Table 1). This attenuation of myocardial dysfunction in db/db mice was accompanied by a protection from ischemia reperfusion injury as evidenced by reduced infarct size (Figure 1f, g) and significantly improved functional recovery post-myocardial infarction vs untreated db/db mice (Figure 1h).

### AZD treatment improves cardiac metabolism in dbCM

**Figure 2.**
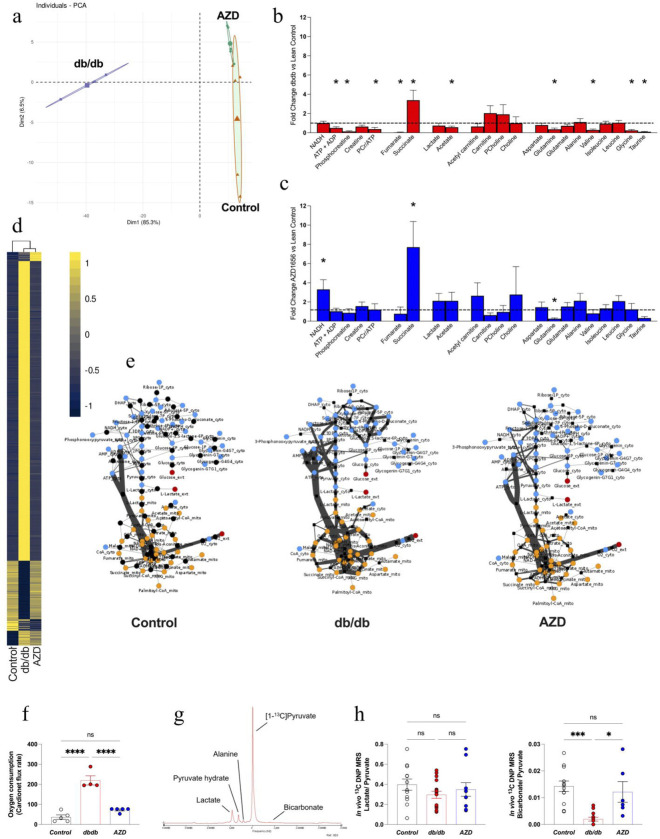
AZD treatment improves cardiac metabolism in dbCM **a**) PCA plot of ^1^H NMRs metabolomic profiling **b**) Fold change db/db vs control ^1^H NMR metabolomic spectroscopy profiling, n=6/group **c**) Fold change AZD treated db/db vs control ^1^H NMR metabolomic spectroscopy profiling, n=6/group **d**) Cardionet metabolic flux balance analysis based on the ^1^H NMR metabolomic profiling and plasma metabolomic analysis (n=6/group) **e**) Visualization of CardioNet metabolic flux predictions using Cytoscape. Metabolites and reactions are depicted as nodes and lines, respectively. The line thinckness corresponds with the calculate flux rate. **f**) Prediction of oxygen consumption rates based on CardioNet simulations. **g**) Representative annotated spectrum from *In vivo* ^13^C DNP MRS cardiac metabolic flux assessment **h**) *In vivo* ^13^C DNP MRS measured TCA cycle flux (Control n=12, db/db n=16, AZD n=10). Multiple group comparison by ANOVA. Two-tail comparison by student t-test. *P<0.05 ***P<0.01 ****P<0.001

Untreated db/db mice were characterized by severe cardiac metabolic dysfunction (Figure 2). Principal component analysis of the metabolomic profile, assessed by high-resolution ^1^H NMR spectroscopy (Figure 2a) confirmed that T2D leads to a distinct metabolomic profile of db/db hearts versus controls. Specifically, the metabolomic profile of T2D mice is characterized by a significant depletion of amino acids (glutamine, valine, glycine, taurine) and acetate, as well as altered TCA cycle intermediates (depleted fumarate, elevated succinate, Figure 2b). Collectively, metabolomic changes in db/db hearts were associated with reduction in energy reserves (phosphocreatine, ATP+ADP, PCr/ATP+ADP, Figure 2b).

In order to further analyse the cardiac metabolic profile in db/db mice, we applied a systems biology approach that combines metabolomics with constrained-based *in silico* modelling.^32,43^ To infer flux distributions, we integrated targeted metabolomics data from control, db/db and AZD-treated groups into flux balance analysis (FBA). Metabolic flux distributions were calculated using the mammalian network of cardiac metabolism, CardioNet^44^ based on quantified fold changes of metabolite concentrations between control and experimental groups as well as circulating metabolic profile. CardioNet *in silico* flux balance analysis of db/db cardiac metabolism further confirmed a markedly different metabolic profile compared to controls (Figure 2d). Computational simulations of db/db mice revealed enhanced anaerobic glycolysis and glucose flux through the pentose phosphate pathway, while glucose oxidation in the Krebs cycle was decreased (Figure 2e). The shift in extracellular nutrient supplies enhanced lipid oxidation (Figure 2e), which increased reactive oxygen species generation (Figure 2e) and markedly elevated oxygen consumption (Figure 2f). Moreover, *in vivo* metabolic flux assessment using ^13^C hyperpolarized magnetic resonance (Figure 2g) showed that *db/db* mice had 95% lower cardiac pyruvate dehydrogenase flux *in vivo*, reflecting a reduction in cardiac glucose oxidation (Figure 2h).

Remarkably, treatment with AZD ameliorated cardiac metabolic dysfunction in db/db hearts. PCA analysis showed that the metabolomic profile post-drug treatment was indistinguishable between AZD1656-treated db/db hearts and controls (Figure 2a Supplementary Figure 2). The ^1^H NMR metabolomic profile was comparable between drug-treated db/db and control hearts (Figure 2c) short of persistent supra-normal elevation of succinate, an increase in NADH and a reduction in glutamine concentration. *In silico* modelling also showed improvements in cardiac metabolic flux reactions (Figure 2d), including markedly improved lipid oxidation, ROS production and hexosamine pathway utilization (Figure 2e). Our CardioNet flux analysis (Supplementary Data 1) predicts that AZD treatment results in a marked reduction in anaerobic glycolysis leading to a reduction in lactate release compared to db/db hearts. One of the most notable post-drug treatment adaptations is the striking 2-fold reduction in CardioNet-calculated oxygen consumption (Figure 2f) returning it to near-control rate. Furthermore, ^13^C hyperpolarized magnetic resonance demonstrated that *in vivo* cardiac metabolic flux was improved (Figure 2h), showing increased conversion of [1-^13^C]pyruvate to ^13^C-bicarbonate, indicative of increased PDH flux. Of note, in healthy hearts, acute AZD treatment alone did not exert any metabolic or functional effects (Supplementary Figure 3).

**Figure 3.**
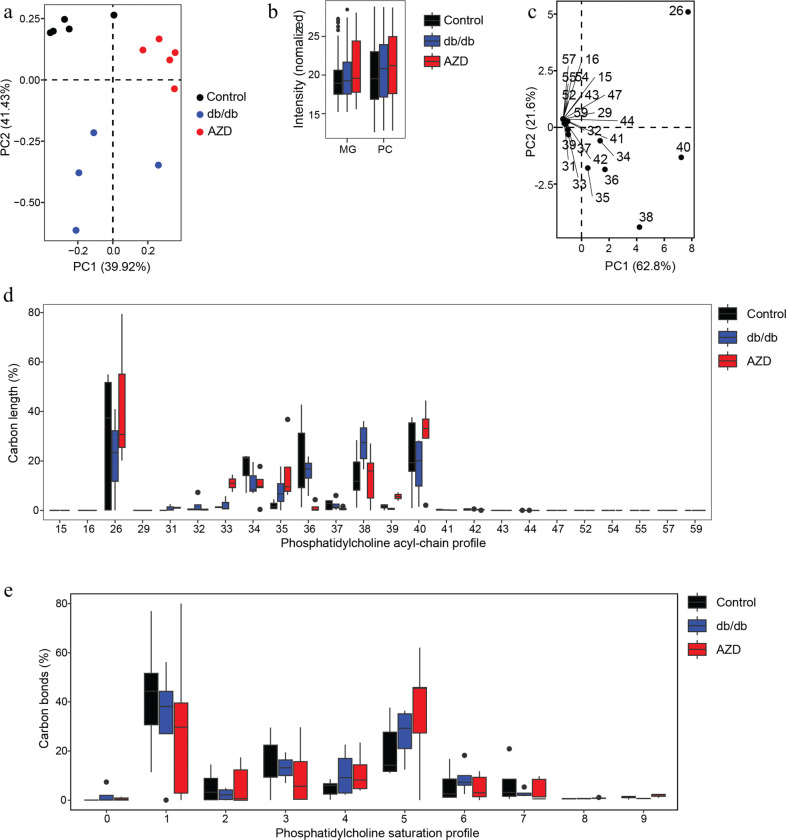
AZD treatment improved cardiac lipidomic profile **a**) Partial least square-discriminant analysis (PLS-DA) visualization of lipid abundances. Samples (n=5/group) are grouped according to biological replicates and dimensions (Dim) 1 and 2 together captured 81.35% of variability. **b**) Contribution of significantly altered phospholipid (PL) classes across biological samples. MG, Monoacylglycerols; PC, phosphatidylcholine **c**) Principal component analysis (PCA) of PC acyl chain profiles. Total carbon lengths were compared across biological samples. Dimensions 1 and 2 captured 84.4% of variability. PC(40) and PC(26) were the strongest contributors to PC alterations in treatments. **d-e**) Average total chain length and degree of saturation varied between experimental groups.

To further study the impact AZD on cardiac lipid metabolism, we comprehensively quantified the distribution and abundance of 13 major lipid classes in heart tissues by non-targeted MS/MS lipidomic analysis (Figure 3, Supplementary Data 2). We analysed lipidomics datasets by supervised multivariate classification using partial least square-discriminant analysis (PLS-DA).

Among treatment-dependent separation, 81% of the total variance was captured in the first two dimensions (Figure 3a). Phosphatidylcholine (PC) and monoacyl-glycerols (MG) showed significant alterations across all three experimental groups (Figure 3b). Within the fatty acyl (FA) pools of PC, the most influential summed acyl chain lengths were identified by principal-component analysis (PCA, Figure 3c), which showed that FA26, FA40, and F38 were dominant. Additionally, FA34, 35 and 36 contributed to PC variability. Thus, we analyzed the PC fatty acyl incorporation and double bond distribution across all experimental groups and found that db/db mice had decreased FA26 and FA40 abundance, while FA38 was upregulated compared to control animals. In contrast, treatment with AZD reversed these FA profiles towards control levels by reducing the overall contribution of FA38 and increasing the abundance of FA26 and FA40. A major component of PC across experimental groups was FA26, which contributed more than 40% of the total pool. These alterations were also reflected in the distribution of double bonds within the PC class. Both db/db and AZD increased the saturation of PC species from mono-unsaturated towards 4 to 5 double bonds. Untreated db/db mice had the longest and most unsaturated PC acyl chains (Figure 3d). In contrast, treatment with AZD caused less saturated and shorter acyl chains. The di-unsaturated to mono-unsaturated ratio was typically lower in untreated db/db hearts (0.07) compared to AZD treated animals (0.2) and controls (0.129).

### AZD treatment effects are not mediated by changes in systemic metabolism

**Figure 4.**
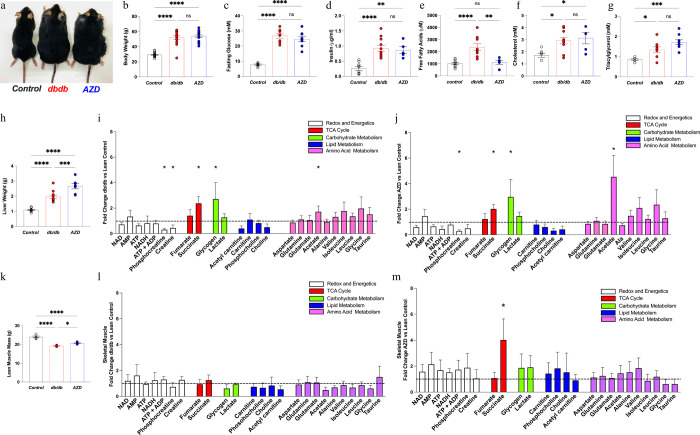
The systemic effect of AZD treatment in db/db **a**) Representative images of control, db/db and AZD treated db/db mice from the study **b**) Body weight (Control n=23, db/db n=17, AZD n=20) **c**) Fasting plasma glucose (n=9/group) **d**) plasma insulin (Control n=10, db/db n=11, AZD n=6) **e-g**) circulating lipid metabolism constituents [free fatty acids (Control n=11, db/db n=9, AZD n=5), cholesterol (Control n=7, db/db n=10, AZD n=5), triacylglycerol (Control n=7, db/db n=11, AZD n=9). Liver phenotype profiling **h**) Liver weight (Control n=12, db/db n=11, AZD n=7) **i**) Fold change db/db vs control ^1^H NMR metabolomic spectroscopy profiling **j**) Fold change AZD vs control ^1^H NMR metabolomic spectroscopy profiling (Control n=6, db/db n=7, AZD n=7) Skeletal Muscle phenotype (Control n=6, db/db n=6, AZD n=5) **k**) lean muscle mass by body composition analysis (n=5/group) **l**) Fold change db/db vs control ^1^H NMR metabolomic spectroscopy profiling, **m**) Fold change AZD vs control ^1^H NMR metabolomic spectroscopy profiling, (n=5/group) *P<0.05, **P<0.01, ***P<0.001 multiple group comparison by ANOVA, two-tail comparison by student t-test. *Note:* Parameters measured over the course of 6-year study in multiple cohorts thus sample size varied.

In order to examine whether the AZD treatment driven cardiometabolic improvements were a result of systemic metabolic alterations in db/db animals (Figure 4a), extensive analyses of blood, liver, adipose tissue and skeletal muscle were performed. AZD treatment did not improve obesity (body weight, Figure 4a, b), hyperglycaemia (Figure 4c, Supplementary Figure 4), hyperinsulinemia (Figure 4d) or insulin resistance (adiponectin, Supplementary Figure 4). Whilst AZD treatment significantly reduced the circulating free fatty acid concentration (Figure 4e), the remnant circulating metabolite profile was not improved (Figure 4 f,g, Supplementary Figure 4). AZD treatment did not improve the diabetic liver phenotype (Figure 4h, alanine transferase, alkaline phosphatase, Supplementary Figure 4) or metabolism (Liver ^1^H NMRs metabolomics Figure 4i,j, Supplementary Figure 5a). AZD treatment led to a minute increase in lean muscle mass vs db/db (Figure 4k) but had limited effect on the skeletal muscle metabolome (Figure 4 l, m, Supplementary Figure 6b). Furthermore, treatment with AZD did not reduce whole body fat mass (Supplementary Figure 6) nor alter the lipid composition of the adipose tissue (Supplementary Figure 6).

### AZD treatment reduces T-cell mediated cardiac inflammation and fibrosis in dbCM

**Figure 5.**
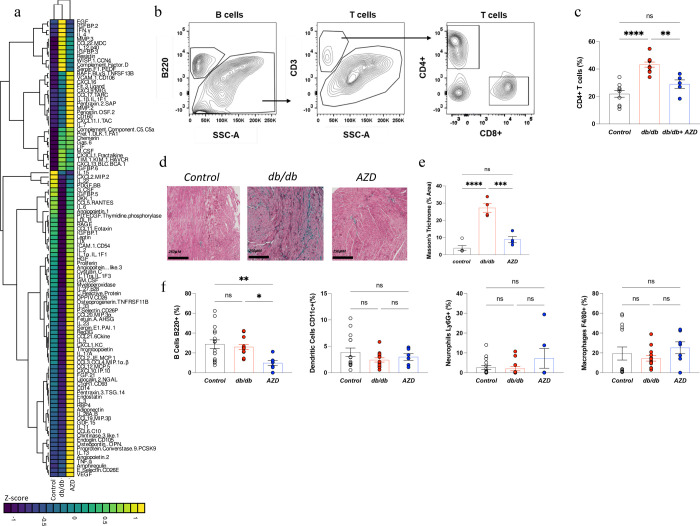
AZD treatment reduces T-cell mediated cardiac inflammation and fibrosis in dbCM **a)** Circulating XXL cytokine plasma panel heatmap summary (Control n=10, db/db n=12, AZD n=10 Representative FACS contour plots **c**) Relative frequency of myocardial CD4+ T cells quantified by FACS **d**) Masson trichrome stained representative cardiac cross sections **e**) quantification of Masson trichrome fibrosis staining intensity (Control n=5, db/db n=4, AZD n=4) **f**) Relative frequency of B cells, dendritic cells, neutrophils and macrophages *P<0.05, **P<0.01, ***P<0.001.Multiple group comparison by ANOVA.

Untreated db/db mice were characterized as having low-grade systemic inflammation, observed from alterations to the circulating cytokine profile (Figure 5a, Supplementary Figure 7), with the most abundant inflammatory factors being MMP3, Serpin F1 PEDF, EGF, Complement factor D, IFNγ, PDGF.BB, GDF 15, FgF21, serpin E1, IL1, myeloperoxidase, leptin, osteoprogenerin, IGFBP1, LDL-R. In addition to the systemic inflammatory milieu, cardiometabolic and functional derangements in db/db mice were concomitant with unregulated T cell-mediated cardiac inflammation (Figure 5b,c) and fibrosis (Figure 5d,e). In terms of infiltration of other inflammatory cell types, there was no evidence of increased infiltration of B cells (B220), Ly6C-lo monocytes, CD11c+ dendritic cells, neutrophils Ly6G+ or macrophages F4/80 (Figure 5f) in db/db hearts.

AZD treatment improved cardiac inflammation in db/db mice. The drug improved the compromised cardiac immunopenetrance, as in treated db/db mice there was a reduction in myocardial CD4+ T cell infiltration (Figure 5c) and an improvement in cardiac fibrosis (Figure 5d,e). AZD had no effect on migration of dendritic cells, neutrophils or macrophages (Figure 4f) however, it did cause reduction in myocardial B cell infiltration (Figure 5f). In terms of soluble cytokine profile, there was a marked change with AZD treatment (Figure 5a, Supplementary Figure 7), with significantly altered concentrations including serpin E1/PAI-1, angiopoietin-2, CXCL16, CCL20/MIP-3a, CXCL1, FGF21, BAFF/Blys/TNFSF13B, IL-12 p40, GDF-15, FGF acidic (Figure 5a, Supplementary Figure 7).

### Treatment with AZD normalizes the expression of genes regulating key intracellular pathways

**Figure 6.**
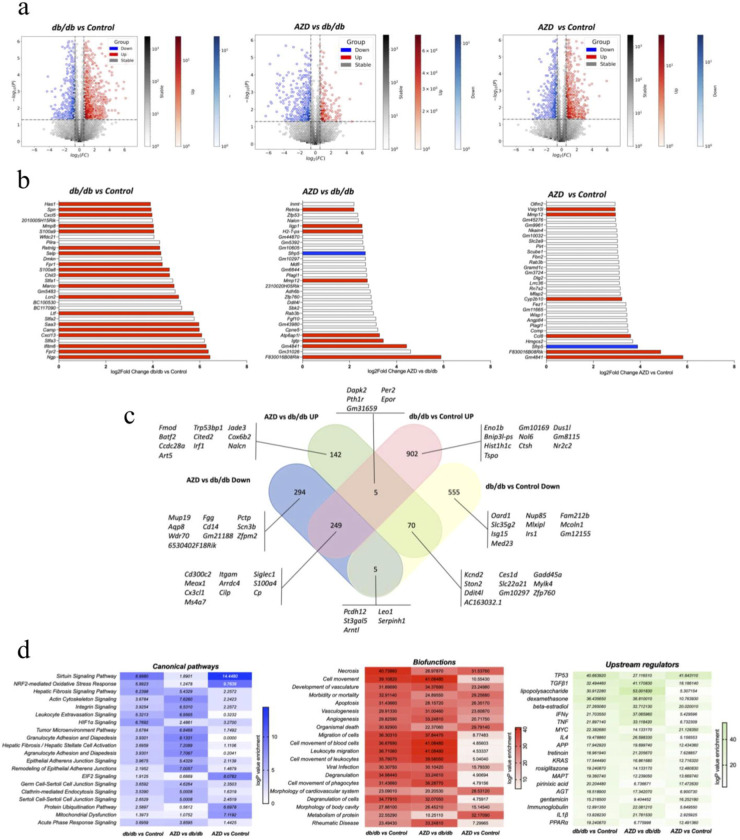
Treatment with AZD improves the expression of genes regulating key intracellular pathways Visualisation of differentially expressed genes (DEGs) using hexbin plot summaries. X axis values are log2fold Change vs y axis − log 10 (P value) of **a**) db/db vs Control, AZD vs db/db, AZD vs Control. Y axis vertical cut off log2 (1.5) ± 0.585 and x axis horizontal cut off -np.log10(0.05) ~ 1.301 **b**) Top 30 most upregulated DEG in db/db vs Control, AZD vs db/db, AZD vs Control. Red bars pro-inflammatory regulators, blue bar- anti-inflammatory regulators. **c**) The Venn diagram of overlapping homologous DEGs among the three data sets (Control, db/db and AZD). Threshold was set to be FC > 1.5 (or <1/1.5) and P < 0.05. Sample genes ( increased and decreased) from each of the subsets are given: abs_diff_log2FC = abs(log2FoldChange_AZ_vs_dbdb − log2FoldChange_dbdb_vs_CTRL) **d**) Heatmap plots of − log p value of DEGs resulting from hypergeometric test and Benjamini-Hochberg p-value correction to identify all ontology and pathway terms that contain a greater number of genes in common with an input list than expected by chance using Qiagen IPA software^41^. This is expressed as a − log p value in the heatmaps. All RNA seq data presented in this figure is based on n=6 hearts/group.

Development of dbCM in db/db mice was characterized by significant alterations in cardiac gene expression [1379 differentially expressed genes (DEGs) db/db vs control, 503 genes db/db vs AZD, 1480 genes Control vs AZD, Figure 6a]. G profiler gene enrichment analysis (Supplementary Figure 8) shows dbCM caused extensive alterations in the expression of genes governing key aspects of cardiac function: molecular functions, biological processes, cellular components, protein complexes as well as biological pathways. Analysis of the 30 most upregulated genes in db/db vs control hearts shows that 19 out of the top 30 over-expressed genes are pro-inflammatory mediators (Figure 6b). Further Venn diagram analysis (Figure 6c) identified overlapping homologous DEGs of db/db, control and AZD-treated db/db hearts. The Venn diagram analysis of gene homology between the groups highlights the upregulation of the inflammation mediating genes (*Frp2, Ifitm6, Camp, Ltf, Ngp, Saa3*) including leukocyte migration mediator *Selp* in db/db versus lean controls (Figure 6c). Pathway analysis further identified genetic hallmarks of dbCM and has shown the upregulation of canonical pathways, biofunction regulators and upstream regulators (Fig 6d) of inflammation, apoptosis, T-cell migration/infiltration, inflammatory signalling and metabolism (fats and carbohydrates) (Supplementary Figures 9–11).

AZD treatment of T2D markedly changed the altered gene expression profile of T2D hearts (Figure 6 a,b) as treatment reversed pathologically high gene expression in db/db hearts compared to untreated (503 genes, AZD vs db/db Figure 6b). Out of the 30 most expressed DEGs in AZD vs db/db hearts and AZD vs Control hearts only 8 (AZD vs db/db) and 6 (AZD vs Control) are pro-inflammatory. Of note, AZD treatment also increased expression of *Sfrp5* (frizzled-related sequence protein 5), a mediator of protection against inflammation and apoptosis via inhibition of Wnt5a/JNK pathway^45^. AZD treatment reduced the number of shared, upregulated inflammation mediators in AZD versus Control to only 3 genes (Venn diagram, *GM4841, F830016B8Rik, Sfrp5*, Figure 6c). Pathway analysis reveals that drug treatment resulted in the opposite pattern of myocardial pathway gene expression: for instance, the allograft rejection pathway that showed the biggest increase in log fold change in expression in db/db vs control is the least expressed in db/db vs AZD (Supplementary Figure 8). These gene expression changes driven by AZD treatment also included a reduction in the expression of genes in reactive oxygen species production pathways, metabolism of lipids and multiple inflammation mechanisms and signals (Figure 6d).

In addition, treatment with AZD targeted and improved a whole series of pathways involved in biological functions (Figure 6d) including necrosis, cell movement, allograft rejection, apoptosis, leukocyte migration, signalling pathways (sirtuin, Nrf2, hepatic signalling, leukocyte migration and signalling, Figure 6d, Supplementary Figure 8). Furthermore, AZD treatment impacted a range of upstream regulators including improvement of pro-inflammatory signals (TGFβ1, IFNγ, IL4, PPARα Supplementary Figure 8).

Crucially, the mechanistic impact of the AZD treatment on db/db hearts was shown by complete amelioration of the dysregulated leukocyte extravasation signaling governing T-cell infiltration of the myocardium (change of signaling pathway intermediate components from red to green, detailed Kegg pathway in Supplementary Figure 9) as well as of two key metabolic regulator pathways: HIF1α (Supplementary Figure 10) and NRF2-mediated Oxidative Stress Response (Supplementary Figure 11).

## Discussion

**Figure 7.**
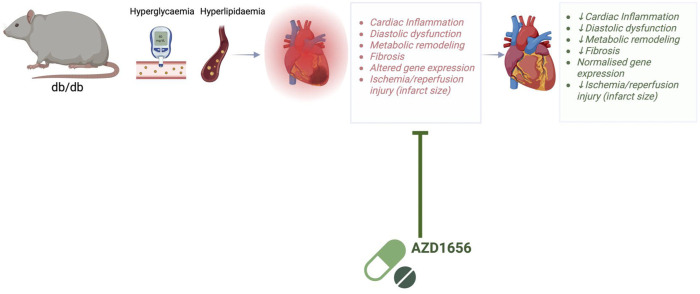
Summary of the key study findings AZD treatment may represent a new therapeutic concept for the treatment of dbCM-the use of small molecules targeting immunometabolism to reduce cardiac inflammation in the diabetic heart. Figure generated with Biornder.com

Given the previously proposed immunomodulatory effect of AZD^8,26^, we investigated the possibility that this treatment would improve cardiac inflammation and in turn attenuate cardiac remodelling in dbCM. Systemic T-cell mediated inflammation has been extensively phenotyped in T2D^46^ and other chronic metabolic stress states^2,47^, however cardiac inflammation in T2D remained to be identified and profiled. As previously observed, 20-week old db/db mice were defined by the phenotypic features representative of human dbCM: obesity, hyperglycaemia and impaired diastolic function (*in vivo* and *ex vivo*) (summarised in Figure 7). Analysis of 20-week db/db diabetic hearts showed extensive autoimmunity by T-cells accompanied by an increase in systemically altered pro-inflammatory cytokine production and fibrosis, which was concomitant with altered cardiac metabolism: demonstrated through *in vivo* substrate utilisation (hyperpolarized ^13^C magnetic resonance spectroscopy), energetics, oxidative phosphorylation, and amino acid metabolism (^1^H NMR spectroscopy).

Remarkably, 6-weeks of pharmacological treatment with AZD attenuated the functional and metabolic remodelling observed in the untreated diabetic heart, resulting in improved post-ischemic functional recovery and reduction in ischemic damage.

One of the key challenges in dbCM is the loss of metabolic flexibility^48^. However, AZD drug treatment achieved significant improvements in the cardiac metabolomic profile and flux as observed from both *in vivo* and *in silico* metabolic assessment. These changes are suggestive of improved substrate utilization flexibility. Furthermore, AZD pharmacological intervention in db/db mice resulted in a 68% reduction in cardiac oxygen consumption. Increased myocardial oxygen consumption and O_2_ wasting due to uncoupled mitochondrial function have been shown to reduce cardiac efficiency in T2D ^49,50^. Thus, the reduction in oxygen consumption post-AZD treatment is critical for the improvement in cardiac function and for the reduction in ischemia-reperfusion damage. dbCM in db/db mice was also characterized by differences in the fatty acyl chain composition of glycerolipids such as phosphatidylcholine as well as monoacyl-glycerols. AZD treatment significantly improved the cardiac phospholipid composition suggestive of improved lipid metabolism as well as cellular membrane and mitochondrial integrity^51 52^. Our studies demonstrated that pharmacologic interventions that attenuate obesity-induced alterations in phospholipid composition and homeostasis positively impact cardiac metabolism and function. Bulk cardiac tissue RNA sequencing analysis showed that AZD treatment significantly improved the gene expression profile including genes governing key metabolic and inflammatory response pathways (ie. HIF1α, PGC1α, apoptosis, necrosis, leukocyte extravasation, fatty acid metabolism, oxidative phosphorylation).

Experimental AZD treatment outcomes could have been achieved via two potential mechanisms of action: by improved glycaemic control (original drug indication) and/or by modulation of the immune response^8^. Since no differences in glycaemic control were detected between groups, it is unlikely that the differences in outcomes were a result of the glucose lowering effect of AZD.

Furthermore, systemic metabolomic profiles of blood, liver, adipose tissue, skeletal muscle were largely unaffected thus any beneficial effects observed in db/db treated hearts were not driven by marked improvements in systemic peripheral tissue metabolism (liver, muscle). We have, however, observed a significant reduction in the circulating fatty acid concentration with an accompanying increase in body fat content post-AZD treatment. This could have contributed to the improvement of myocardial metabolism and inflammation.

Reduced circulating fatty acids would have affected myocardial metabolism, specifically PDH flux *via* fatty acid regulation of PDK4^53,54^ and reduced systemic pro-inflammatory T-cell proliferation^55^. Fatty acid oxidation is downregulated upon T cell activation and influences immune checkpoint protein regulation^56^. T-cell inflammation has been previously linked with the development of heart failure in both animal disease models and humans^57^. T-cell costimulatory blockade, using the rheumatoid arthritis drug abatacept, significantly reduced the severity of cardiac dysfunction in heart failure. This therapeutic effect occurred via the inhibition of activation and cardiac infiltration of T-cells and macrophages, leading to reduced cardiomyocyte death.^57^ One of the key characteristics of dbCM is an increase in fibroblasts which have been shown to act as antigen presenting cells^58^ driving myocardial T-cell infiltration and inflammation^59^.

The immunophenotyping data here suggested that AZD treatment induced an immunomodulatory effect in db/db mice via a reduction in myocardial T-cell and B-cell penetrance as well as by reducing fibrosis. This is consistent with rebalancing of the immune response observed in the ARCADIA trial in COVID19 T2D patients^8^. In terms of the circulating inflammatory milleu, the alteration in the peripheral chemokine data suggested changes in soluble rather than membrane bound chemokines cleaved by MMP9 which also increased with AZD. The resultant change in chemokine profile post-drug treatment could potentially inhibit inflammatory responses, as they block their binding to membrane bound chemokines. For instance, an alteration in circulating IL-12 is indicative of less T-cell effector function and DC activation^36^.

The ARCADIA trial outcome has introduced a novel immunometabolic therapeutic concept: pharmacological targeting of the endogenous immune cells to turn them into the therapeutic agents within the body^8^. Previously, this drug development approach, whether it was used in exogenously treated or engineered cells has failed^8,60–62^. Our study results contribute to the growing field of cardio-immunology as they suggest that AZD could be used as a novel immunomodulatory drug to attenuate cardiometabolic dysfunction in type 2 diabetes as well as the whole host of sterile-inflammation pathologies.

## Supplementary Material

Supplement 1

## Figures and Tables

**Table 1:** Morphological characteristics of the experimental model and impact of AZD treatment on invivo function assessed by Cine MRI and Echocardiography

	Control	db/db	AZD

***Morphological Characteristics***			

Wet heart weight (g)	0.15±0.007	0.16±0.007	0.16±0.008
Wet heart weight : tibia length	0.08±0.006	0.12±0.02	0.08±0.007
Wet : dry lung weight	6.1±1.1	6.4±0.7	6.6±1.0

***Cine-MRI***			

LV Mass (mg)	104.5±3.7	88.1±2.6**	86.3±3.3**
End diastolic volume (μL)	66.1±2.6	59.3±1.5	5 8.1±3.2
End systolic volume (μL)	25.4±1.6	18.7±1.2**	16.0± 1.3***
Ejection Fraction (%)	61.9± 1.4	68.4±1.9*	71.7±2.8**
Stroke Volume (μL)	40.6±1.5	40.6±1.4	42.1±3.3
Cardiac output (mL/min)	16.3±0.5	14.2±0.9	15.0± 1.4

***Echocardiography***			

Deceleration Time (ms)	19.2±2.1	32.2±1.5***	27.3±1.7
Myocardial Performance Index	0.50±0.03	0.74±0.08***	0.62±0.04
Fractional shortening (%)	40.8±0.8	40.4±0.8	39.7±1.1
Pulmonary Artery Peak Velocity (mm/s)	-701.8±33.2	-533.0±58.3*	-677.4±38.5
Ascending Aorta Peak Velocity (mm/s)	1930±229	1161±32*	1809±55.8^++^

Morphological Characteristics: wet heart weight (Control n=15, db/db n=15, AZD n=11), wet heart weight:tibia length (Control n=12, db/db n=10, AZD n=10).Cine MRI data (Control n=18, db/db n=15, AZD n=11). Echocardiography: deceleration time (n=5/group), MPI (Control n=10, db/db n=9, AZD n=10), Fractional shortening (Control n=10, db/db n=9, AZD n=10); Pulmonary Artery Peak Velocity (Control n=5, db/db n=3, AZD n=5), Ascending Aorta Peak Velocity (Control n=4, db/db n=3, AZD n=4), Kolmogorov-Smirnov test data normality test, multiple group comparison by one-way ANOVA with Bonferroni’s multiple comparison test. P<0.05, individual P values indicating level of significance stated in the table column (P value).

** P<0.005 db/db vs control

*** P<0.0005 db/db vs control

++ P<0.05 AZD vs db/db

## Data Availability

RNA sequencing data is available (open access) on Array Express accession E-MTAB-13849. All raw data deposited on Dryad (open access).
